# Forensic NMR metabolomics: one more arrow in the quiver

**DOI:** 10.1007/s11306-020-01743-6

**Published:** 2020-11-07

**Authors:** Emanuela Locci, Giovanni Bazzano, Alberto Chighine, Francesco Locco, Ernesto Ferraro, Roberto Demontis, Ernesto d’Aloja

**Affiliations:** 1 Department of Medical Sciences and Public Health, Section of Legal Medicine, University of Cagliari, Cagliari, Italy; 2 Department of Medical Sciences and Public Health, Legal Medicine Section, University of Cagliari, Cittadella Universitaria di Monserrato, 09042 Monserrato, CA Italy

**Keywords:** ^1^H NMR, Metabolomics, Forensics, Biological traces, Post-mortem interval, Drug abuse

## Abstract

**Introduction:**

NMR metabolomics is increasingly used in forensics, due to the possibility of investigating both endogenous metabolic profiles and exogenous molecules that may help to describe metabolic patterns and their modifications associated to specific conditions of forensic interest.

**Objectives:**

The aim of this work was to review the recent literature and depict the information provided by NMR metabolomics. Attention has been devoted to the identification of peculiar metabolic signatures and specific ante-mortem and post-mortem profiles or biomarkers related to different conditions of forensic concern, such as the identification of biological traces, the estimation of the time since death, and the exposure to drugs of abuse.

**Results and Conclusion:**

The results of the described studies highlight how forensics can benefit from NMR metabolomics by gaining additional information that may help to shed light in several forensic issues that still deserve to be further elucidated.

## Introduction

Forensic science represents the application of several scientific disciplines, such as medicine, chemistry, physics, and biology to the sphere of criminal investigation. In a more confined context, like the legal medicine scenario, it is mainly focused in giving evidences to be used either in the criminal or in the civil justice system through the forensic implementation and validation of scientific methods originally developed for different purposes.

In the last decades, forensic science addressed several issues, which in the previous period were an unmet need, due to the inadequacy of the available scientific tools. Challenges such as human individualization, toxicological analysis on different biological specimens, and reproducible evaluation of pathological findings during autopsy have been solved by the introduction of DNA profile analysis on smaller and smaller stains (Prinz and Lessig 2014), by the development of Gas Chromatography (GC), Liquid Chromatography (LC), and Capillary Electrophoresis (CE) combined with Mass or Tandem-Mass Spectrometry (MS or MS/MS) methods for quantification of several drugs and their metabolites in human biological fluids (BFs) (Gottardo et al. 2012; Drummer and Gerostamoulos 2014), and by the implementation of advanced imaging tools in the identification of the cause of death, such as Computed Tomography with or without contrast medium and Magnetic Resonance Imaging (Ampanozi et al. 2020), and Optical Coherence Tomography (Nioi et al. 2019).

Although the incredible scientific advancement in this field, the appearance of ‘-omics’ sciences represents a unique opportunity to deal with old issues from a completely new point of view. Among them, genomics and transcriptomics have been the first to be applied in the forensic arena. In the last years, a huge effort was made by the forensic community to unravel the genetic basis of several legal conundrums, such as human/animal stains identification or the hereditary network underlying some causes of death, e.g. Sudden Infant Deaths (SIDs), Sudden Unexplained Death in Epilepsy (SUDEP), arrhythmogenic deaths—among them, Brugada Syndrome (BS), Long QT Syndrome (LQTS), Short QT Syndrome (SQTS), Cathecholaminergic Polymorphic Ventricular Tachycardia (CPVT) (Oliva et al. 2011; Coll et al. 2016; Heathfield et al. 2018), and post-mortem interval (PMI) estimation by means of gene expression patterns (Zhu et al. 2017) or human tissues transcriptomes (Ferreira et al. 2018).

Metabolomics represents the quali-quantitative study of the low molecular weight metabolites present in a biological system and provides information on the metabolic modifications induced by any external factor. Depending on the information required, metabolomics allows to perform a targeted analysis or to obtain a global profile of the metabolome. A major advantage of metabolomics in forensics is represented by the possibility of integrating data originating from different body compartments or BFs (Castillo-Peinado and Luque de Castro 2016). When NMR metabolomics is considered, further advantages include that NMR is extremely versatile, robust, non-destructive, non-selective, and highly reproducible.

Metabolomic studies applied to forensic medicine cover different topics: the identification of BFs traces; the study of the tissues and BFs metabolomic profile modifications after death with the aim of estimating the PMI; the toxicological analysis of human BFs in order to detect both drugs of abuse and their biological active metabolites, and to identify specific metabolomic pathways related to their acute or chronic intake; the possible metabolomic effects of forensic relevant pathological insults (*noxae*) either on tissues or BFs; and the correlation of human microbiome with physiological and pathological conditions. These studies have been described in some relatively recent review papers (Zapata et al. 2015; Castillo-Peinado and Luque de Castro 2016; Santos et al. 2018; Dinis-Oliveira 2019; Steuer et al. 2019).

In this review we exclusively focused our attention on the use of NMR as analytical platform for forensic metabolomic studies. The literature was extensively analysed in order to depict the latest state of the art in all the forensic areas in which NMR metabolomics was introduced as a new or alternative analytical tool.

## Forensic analysis of body fluids

Identification of BF traces in a crime scene plays a pivotal role in forensic science. The main steps for the identification of a trace are the characterization of the BF type, the species (human or animal) identification, and the personal identification. While the two latter are usually addressed with DNA analysis, the BF identification in single or composite traces still represents a complex, although sometimes forgotten, task. Traditional, and sometimes very old, presumptive tests are still used, such as Kastle–Meyer and Luminol for blood and alpha-amylase for saliva (Prinz and Lessig 2014), but they are often inaccurate and specific for only one biological matrix. The main disadvantage of these tests is their destructive nature, which results in potential partial sample loss for subsequent DNA analysis. Besides, new tools such as mRNA, miRNAs, and methylation patterns are nowadays available for BF typing. The ideal BF assay should give a unique fingerprint for each BF, while being reproducible, fast, highly sensitive/specific, non-destructive, and confirmatory.

The ^1^H NMR spectrum of a BF gives a snapshot of the global profile of all the detectable low molecular weight metabolites present in a sample in a single experiment. It has the advantage of relying not on a single chemical feature but on a fingerprint composed by a multitude of chemical features. Furthermore, it is rapid, it does not need extensive sample preparation and it is non-destructive, meaning that samples are completely recovered after the analysis and available for further investigations. The main limit of NMR is represented by its intrinsically low sensitivity which causes a problem when dealing with small sample volumes or very low metabolite concentrations. This could have a high impact on forensic studies for solid identification of low concentration metabolites, or metabolites obscured by the water peak or large macromolecules. In such a scenario, rigorous application of the MSI minimum reporting standards for chemical analysis is mandatory (Sumner et al. 2007). In any case, at least a MSI level 2 identification should be requested to let the NMR analysis be presented as a biological proof in a legal context, and confirmation with a different analytical platform could be requested.

^1^H NMR spectroscopy has been widely used to characterize the molecular compositions of BFs for clinical purposes (Lindon et al. 2000), and exhaustive datasets listing human urine, serum, and CSF compounds, their concentrations in the healthy population, and their known disease association, have already been published in the literature (Psychogios et al. 2011; Bouatra et al. 2013; Wishart et al. 2018). Protocols for sample preparation and analysis of these BFs have been reported (Beckonert et al. 2007; Emwas et al. 2019; Bliziotis et al. 2020). Other BFs of forensic interest, such as aqueous humour (AH) (Snytnikova et al. 2017a, b), vitreous humour (VH) (Barba et al. 2010), semen (Gupta et al. 2011), vaginal fluid (Bai et al. 2012), and saliva (Bertram et al. 2009), have been characterized by NMR but their global quali-quantitative reference profile is still lacking, especially in the healthy population.

Scano et al. (2013) were the first to introduce the use of a metabolomic approach, based on the use of ^1^H NMR combined with multivariate statistical analysis, for the forensic BFs identification in single and composite traces. In particular, blood, semen, saliva, and urine samples were analysed together with mock mixtures of two different BFs. A total of 69 BFs were collected from 21 healthy donors (13 males and 8 females), 5 blood samples were eluted from tissue stains, in order to test a condition similar to that occurring in a real forensic scenario, and 12 BF binary mixtures were prepared by adding equal volumes of two different BFs. To mimic “street specimens”, the samples were analysed without any pre-treatment, and for example they were not deproteinized in order to keep the whole profile. ^1^H NMR spectra were obtained, and spectral data were submitted to multivariate analysis and to a fitting procedure for BF identification. A PCA model built on the whole data set indicated that the four BFs were clustered in four groups, with the binary mixtures lying between the two corresponding BFs classes. Successively, the 69 samples were divided into a training set and a test set, averaged BF profiles were obtained for the four BFs, and a mathematical fitting procedure was adopted for BF identification in the test samples, including the mixtures. The results, reported in terms of root mean square differences, demonstrated that each BF could be unambiguously identified due to its peculiar metabolomic profile. Moreover, the procedure applied to the BF binary mixtures allowed to identify from a qualitative point of view the two BF components.

^1^H NMR can also be potentially used for species identification, i.e. to discriminate human from animal BFs. Zailer et al. (2017) showed that blood samples from human and animal species (pets and wild animals) are characterized by different ^1^H NMR metabolomic signatures. More recently, Lee et al. (2019) employed ^1^H-NMR metabolomics to find biomarkers that can be used for the discrimination of human from animal (rat, pig, horse, cat, dog, cow, monkey) urine specimens. Samples showed different patterns in metabolites profile and several peculiar metabolites were identified. The main aim of this work was to identify the sample origin when urine is collected for drug test, as some drug abusers, to skew the analysis results, are used to submit animal urine instead of their own.

As a whole, the ^1^H NMR metabolomics approach proved to be a reliable, specific, flexible, and multi-purpose tool, whenever an unknown stain is found on the crime scene or on fabrics/tissues/objects related to a crime. The main advantages seem to be its ability to guarantee a general unknown approach and the possibility to unravel BF admixtures within a single experiment (so being time saving and allowing a better stain preservation). Major limitations are represented by its low sensitivity, as metabolites are identified in the micromolar range, and this could represent a problem for low concentration metabolites. However, it is worth noting that NMR identification of BF traces would represent only a first level screening analysis to be corroborated by specific confirmatory methods. In this context, the non-destructivity of NMR, and the possibility to submit the recovered sample to a confirmatory (often destructive) analysis represents a key feature. As a whole, whenever the results do not satisfy the standard protocols for identification an inconclusive result should be reported to the court. The scarce availability of NMR equipment in Forensic Departments/Institutes and the limited number of papers published so far represents another limit. Beside all the new ‘omics’ technologies applied by forensic scholars, NMR metabolomics may represent one more analytical approach to be used in this field, but a multicentric and collaborative effort is needed to validate it before its implementation in forensic daily activity.

## Metabolome modifications after death

The estimation of the time since death or post-mortem interval (PMI) remains a challenging issue in the forensic scenario, being one of the most important concerns for forensic pathologists (Madea 2005). Traditional PMI estimation methods are mainly based on the evaluation of a single or few parameters, lacking to some extent precision, accuracy, and reproducibility (Henssge and Madea 2007). In a forensic routine casework, PMI may be inferred by the observation of physical parameters (such as *algor mortis*, *rigor mortis* and *livor mortis*), which, with the only exception of inner and external temperature, are dependent on the examiner individual evaluation (Henssge and Madea 2004). Several biochemical markers, such as potassium, hypoxanthine, sodium, chloride, and calcium have been detected and quantified in different BFs, and the time-related concentration changes have been proposed as a tentative tool for PMI estimation. Even this approach seems to be reliable only in well-established conditions (Madea and Musshoff 2007; Donaldson and Lamont 2013; Swain et al. 2015; Yang et al. 2018). The cause of death, the corpse and the environmental conditions, and several other variables may act as potential confounders playing a relevant role in the modification of the observed biological feature, which may not be solely related to PMI. The analysis of the metabolome modifications, relying on multiple changes of the global profile, seems to be more promising than traditional methods for a reliable PMI estimation. Several works have been devoted to the use of NMR metabolomics of BFs or tissues to monitor the modifications in endogenous metabolites induced by death and to find potential PMI biomarkers (Hirakawa et al. 2009; Rosa et al. 2015; Zelentsova et al. 2016; Snytnikova et al. 2017a, 2017b). Different animal models have been studied and several biological matrices, such as blood, muscles, VH, AH, and cornea have been analysed. Ocular tissues, being anatomically isolated and better preserved from contamination and putrefaction, appear to be the most suitable biological matrices for post-mortem analysis.

The first studies in which NMR spectroscopy was used for forensic purposes appeared in the literature in the ‘80s (Harada et al. 1984). The authors analysed several rat organs and tissues extracts, measured the integrated areas of the major NMR resonances, corresponding to the most representative metabolites, and analysed their modifications during the post-mortem time. They were the first to suggest the use of ^1^H NMR for estimating the PMI.

Almost 20 years later, NMR metabolomics, intended as the combination of the analytical technique with multivariate statistical analysis, was applied to the study of metabolic changes occurring in rat femoral muscles after death (Hirakawa et al. 2009). Samples were collected from 72 rats after 15 min, 1 h, 4 h, and more that 8 h since death, which was caused by suffocation, cocaine overdose, or induced respiratory failure. Femoral tissues were excised and treated with perchloric acid for extraction of the low molecular weight metabolites. Extracted samples were analysed by ^1^H NMR and spectral data submitted to Principal Component Analysis (PCA). In the early post-mortem period, it was possible to statistically differentiate the sample metabolomic profiles according to the cause of death. Besides, the PC scores of the spectral data resulted to be correlated to PMI.

Rosa et al. investigated the post-mortem metabolomic profile modifications in goat VH samples (Rosa et al. 2015). A total of 20 samples were collected at different timepoints: immediately after death, and after 6, 12, 18, and 24 h. A single sample was withdrawn from each eye in order to avoid bacterial contamination due to multiple sampling. Samples were analysed by ^1^H NMR and the spectral data submitted to PCA and Orthogonal Projection to Latent Structures (OPLS) regression modelling. In the PCA model, the samples were distributed along a time-related trajectory, with a two-step behaviour (0–12 h and 12–24 h post-mortem). Glucose was highly represented in samples collected immediately after death, lactate was characteristic of samples collected at 12–18 h after death, and hypoxanthine, myo-inositol, and total glutathione described the score plot region between 18 and 24 h. An OPLS regression model was used to find the metabolites mainly involved in the post-mortem related VH perturbation. The model indicated that lactate, hypoxanthine, alanine, total glutathione, choline, creatine, and myo-inositol, were strongly correlated with time, i.e. they increased at increasing PMI, while glucose and 3-hydroxybutyrate were inversely correlated, decreasing at increasing PMI. The authors corroborate the feasibility of the NMR metabolomic approach to describe the VH post-mortem modifications with the aim of building a model for PMI estimation. In another study, the same authors focused their attention on the topographical composition of goat VH collected immediately after death (Locci et al. 2014). Their results indicated the existence of different functional areas within the VH characterized by a different metabolomic composition. Therefore, caution must be used when VH is collected in the first hours after death, since it is still gelatinous, and composition will depend on the collection site.

The group of Tsentalovich published several papers devoted to the use of metabolomics to investigate the post-mortem changes in both animal and human ocular tissues and biofluids. In a first study a quantitative analysis of the post-mortem metabolomic changes occurring in rabbit serum, AH, and VH was performed (Zelentsova et al. 2016). Samples were collected from 4 animals immediately after death (5–10 min post-mortem) and at several time-points from 1 to 23 h after death, performing multiple sampling from the same eye for AH and VH. Ante-mortem blood specimens were also taken. All the samples were analysed by ^1^H NMR and LC–MS, and metabolite concentrations were determined. For the majority of metabolites, the quantitative results obtained by the two independent techniques were comparable. A comparative analysis of the three BFs indicated that, with only few exceptions, the metabolite concentrations in AH and VH are either similar or lower than those in serum. Among the studied compounds, hypoxanthine, choline, and glycerol were identified as the most promising PMI biomarkers, due to the fact that they changed significantly, monotonously, slowly and with minimal data scattering. Other compounds, including glycine, glutamate, and taurine also showed a monotonous increase with PMI, but the effect was less pronounced. The results indicated that ocular fluids are more suitable for PMI estimation than blood, since the modifications of the latter occurred at a higher rate than in AH and VH. Blood metabolites were significantly modified already 10 min after death, while AH and VH metabolomic modifications proceeded much more slowly and more smoothly, due to the lower metabolic activity, and possibly a delayed microbial metabolomic contribution, of the ocular tissues.

In a following paper, the authors extended a similar approach to the study of human post-mortem AH and blood metabolomic profiles (Snytnikova et al. 2017a). Samples were harvested from 6 cadavers of both sexes, aged 18–81 years, from 5 to 12 h after death. Control samples were obtained from living individuals, patients with advanced cataracts, during the extraction surgery. As in the previous study, samples were quantitatively analysed with the use of two independent methods, ^1^H NMR and LC–MS. The results were mainly focused on describing the differences existing among the post-mortem samples and the samples from living patients. In their following study the authors compared the post-mortem modifications in human AH and cornea (Snytnikova et al. 2017b). Corneal modifications were significantly slower than AH ones. Once again, post-mortem samples were collected at different time-points (from 7 to 24 h after death) but compared as a whole with samples obtained from living individuals undergoing anterior segment surgical procedures, namely cornea replacement for keratoconus. All these studies were mainly focused on investigating the metabolomic alterations occurring after death, but no inferences on the PMI estimation were made.

^1^H NMR metabolomics was applied to investigate mice organ (heart, kidney, liver, spleen, skin, and white adipose tissue) samples collected from ten animals at three different PMI (Mora-Ortiz et al. 2019). Metabolomic alterations were recorded and pairwise Orthogonal Projection to Latent Structure Discriminant Analysis (OPLS-DA) was used to compare two time points for each organ. Kidney, heart, and spleen showed the highest metabolic perturbations, while skin and white adipose tissue were the least altered matrices. Early metabolic modulations were associated with the perturbation of energy metabolism due to the lack of oxygen, while late modulations were associated with microbial metabolism. The results were solely focused on the characterization of the post-mortem modulations and on their biological meaning. Despite the authors introduced the new term “thanatometabolomics” to indicate the proper identification of biomarkers of time since death, they did not propose a model for PMI estimation, and they concluded their discussion highlighting the need of further investigations to accurately determine the time elapsed since death.

^1^H NMR metabolomics was also recently used in a veterinary context to distinguish stillborn calves according to the PMI (Jawor et al. 2019). Although this paper did not directly focus on the determination of time since death, some data may have a relevant interest for forensic purposes. Authors proposed a PLS-DA model—starting from plasma and urine NMR profiles—able to discriminate among four predetermined groups (healthy controls, calves dead in the first 6 h after calving, in utero either with moderate or gross autolytic signs). A set of metabolites, alleged to be correlated with the 3 raw PMI classes, were described, assuming a common cause of death (namely a hypoxic-ischemic event).

Very recently, ^1^H NMR metabolomics data were successfully employed for building a regression model for PMI estimation using AH samples from an ovine model (Locci et al. 2019). A total of 59 samples collected at different times after death up to 24 h (from 118 to 1429 min) were taken from as many eyes, half of which were kept with the eyelids closed and the other half with the eyelids open. The AH metabolomic profile of 38 samples was used to build a multivariate calibration model which was validated with an independent test set consisting of the remaining 21 samples. The prediction error was estimated in the entire temporal window and in three predefined ranges of PMI (less than 500 min, from 500 to 1000 min, and greater than 1000 min). Furthermore, a classification model was built on the 3 PMI classes. The 86% of the test set samples (18 out of 21) were correctly classified as belonging to the correct PMI class. Model interpretation allowed to identify the metabolomic alterations correlated to PMI. Taurine, choline, and succinate resulted to be the metabolites most significantly correlated to PMI. The prediction models individually obtained on these three metabolites resulted to be less accurate than the model obtained using the complete profile, supporting the hypothesis of the higher reliability of a multivariate approach. Concerning the possible effect on the AH metabolite content due to the eye condition (opened or closed), the results indicated that PMI has a stronger effect, and suggested that for the purpose of PMI estimation the eye condition could be in practice neglected. The use of an animals of same sex and age, with an identical mechanism of death, the stability of the experimental conditions (constant humidity and temperature), although ideal conditions for building a reproducible experimental model, represent at the same time the major limitations of this study. “Real world” environmental circumstances may indeed deeply interfere with the post-mortem AH metabolomic profile modifications. Extension of the post-mortem time temporal window would also be desirable.

The power of metabolomics to address the complex issue of PMI estimation lies in the possibility of obtaining the simultaneous profiling of multiple and interacting metabolites, which translates in a greater capability to intercept the post-mortem biological behaviour compared to a single biomarker. Besides, during the post-mortem period, some metabolites are readily consumed, some of them are monotonously accumulated for a relatively long time, while others appear after a certain period, so it would be very difficult to describe such a complex and multiparametric phenomenon by the use of a single predictor.

The majority of the studies addressed to post-mortem metabolome modifications have been carried out using ocular biofluids, since they are the best candidates for this scope. The modifications proceed at a rate that provides the possibility of monitoring them for hours. AH appears to be even more suitable than VH due to the fact that it has a minor content of proteins and lipids and sample preparation is therefore less extensive. The presented studies are difficult to compare because the experimental designs and conditions are different, and several confounding factors, such as the cause of death, the selected individuals or animals, the harvesting of the samples (single or multiple sampling from the same eye) are present. Despite this, some common metabolites were identified to be correlated with the time since death and they can be considered, within a suggestive profile, as potential PMI biomarkers. They are listed in Table 1. The increase or decrease of the single metabolite was not showed because, from the analyses of the reviewed papers, it resulted to be dependent of the experimental design, the studied biofluid, or the temporal window for sample collection. In the post-mortem research, a longitudinal sampling during the observation of the corpse at predefined time-points (at least two sampling, e.g. one from each eye, at a 2–3 h distance) should always be requested to minimise the effects of potential and exogenous variables. If so, it is worth highlighting the strong need for better and better standardization of the experimental protocols in the forensic community and the implementation of a Quality Assurance/Quality Control (QA/QC) programme to be held under the aegis of an international independent agency, as it is currently requested in the accreditation of laboratories of toxicology and/or genetics performing forensic analysis.

**Table 1:** Metabolites related to PMI

Metabolite	Sample	Model	References
Acetate	AH, Cornea, Heart, Kidney, Liver, Serum, Skin, Spleen, Plasma, Urine, VH, WAT	Human, Calf, Goat, Mice, Sheep	Locci et al. (2014, 2019), Snytnikova et al. (2017a, b), Jawor et al. 2019, and Mora-Ortiz et al. 2019
Alanine	Cornea, Kidney, Plasma, Spleen, Serum, Skin, Urine, VH, WAT	Human, Calf, Goat, Mice	Locci et al. (2014), Rosa et al. (2015), Snytnikova et al. (2017a, 2017b), Jawor et al. (2019), and Mora-Ortiz et al. (2019)
Ascorbate	AH, Cornea, Kidney, Serum, Spleen, Urine, VH	Human, Calf, Goat, Mice, Sheep	Locci et al. (2014, 2019), Snytnikova et al. (2017a, b), Jawor et al. (2019), and Mora-Ortiz et al. (2019)
BCAA	AH, Cornea, Heart, Kidney, Liver, Plasma, Serum, Spleen, Urine, VH, WAT	Human, Calf, Goat, Mice, Sheep	Locci et al. (2014, 2019), Snytnikova et al. (2017a, 2017b), Jawor et al. (2019), Mora-Ortiz et al. (2019)
Betaine	Kidney, AH, VH, Serum, Urine	Human, Calf, Goat, Mice, Sheep	Locci et al. (2014, 2019) Snytnikova et al. (2017a), Jawor et al. (2019), and Mora-Ortiz et al. (2019)
Carnitine	Cornea, Heart, Serum	Human, Mice	Snytnikova et al. (2017a, b) and Mora-Ortiz et al. (2019)
Creatine	AH, Cornea, Heart, Kidney, Liver, Plasma, Skin, Serum, Spleen, Urine, WAT, VH	Human, Calf, Goat, Mice, Sheep	Locci et al. (2014, 2019), Rosa et al. (2015), Snytnikova et al. (2017b), Jawor et al. (2019), and Mora-Ortiz et al. (2019)
Creatinine	Cornea, AH, Plasma, Serum, Urine	Human, Calf, Sheep,	Snytnikova et al. (2017a, 2017b), Jawor et al. (2019), and Locci et al. (2019)
Choline	AH, Cornea, Heart, Kidney, Plasma, Serum, Spleen, Urine, VH	Human, Calf, Goat, Mice, Rabbit, Sheep	Locci et al. (2014, 2019), Rosa et al. (2015), Zelentsova et al. (2016), Snytnikova et al. (2017a, b), Jawor et al. (2019), and Mora-Ortiz et al. (2019)
Formate	Cornea, Spleen, AH, Plasma, Urine	Human, Calf, Mice, Sheep	Snytnikova et al. (2017a, b), Jawor et al. (2019), Locci et al. (2019), and Mora-Ortiz et al. (2019)
Glucose	Heart, Kidney, Liver, Plasma, Skin, Spleen, Urine, VH, WAT	Calf, Goat, Mice	Locci et al. (2014), Rosa et al. (2015), Jawor et al. (2019), and Mora-Ortiz et al. (2019)
Glutamate	AH, Heart, Kidney, Liver, Plasma, Serum, Skin, Spleen, VH, WAT	Human, Calf, Mice, Rabbit, Sheep	Zelentsova et al. (2016), Snytnikova et al. (2017a), Jawor et al. (2019), Locci et al. 2019, and Mora-Ortiz et al. (2019)
Glutamine	Heart, Plasma, Serum, Spleen, Urine, VH	Human, Calf, Goat, Mice	Locci et al. (2014), Snytnikova et al. (2017a), Mora-Ortiz et al. (2019), and Jawor et al. (2019)
Glycerol	AH, Cornea, Heart, Serum, Spleen, VH	Human, Goat, Mice, Rabbit	Locci et al. (2014), Zelentsova et al. (2016), Snytnikova et al. (2017a, b), and Mora-Ortiz et al. (2019)
Glycine	AH, Cornea, Serum, VH	Human, Rabbit	Zelentsova et al. (2016), and Snytnikova et al. (2017a, b)
3-OH-butyrate	AH, Urine, VH	Calf, Goat, Sheep	Rosa et al. (2015), Jawor et al. (2019), and Locci et al. (2019)
Histidine	Kidney, Serum, Urine	Human, Calf, Mice	Snytnikova et al. (2017a), Mora-Ortiz et al. (2019), and Jawor et al. (2019)
Hypoxanthine	AH, Cornea, Heart, Serum, Urine, VH	Human, Calf, Goat, Mice, Rabbit, Sheep	Rosa et al. (2015), Zelentsova et al. (2016), Snytnikova et al. (2017a, b), Jawor et al. (2019), Mora-Ortiz et al. (2019), and Locci et al. (2019)
Lactate	AH, Cornea, Heart, Kidney, Plasma, Serum, Skin, Spleen, Urine, VH, WAT	Human, Calf, Goat, Mice, Rat	Harada et al. (1984), Locci et al. (2014), Rosa et al. (2015), Snytnikova et al. (2017a, b), Mora-Ortiz et al. (2019), and Jawor et al. (2019)
Myo-inositol	AH, Cornea, Kidney, Plasma, VH, WAT	Human, Calf, Goat, Mice, Sheep	Locci et al. (2014, 2019) Rosa et al. 2015, Snytnikova et al. (2017a, b), Jawor et al. (2019), and Mora-Ortiz et al. (2019)
Phenylalanine	Heart, Kidney, Liver, Plasma, Serum, Skin, Spleen, Urine, WAT	Human, Calf, Mice	Snytnikova et al. (2017a), Mora-Ortiz et al. (2019), and Jawor et al. (2019)
Pyruvate	AH, Muscle, Plasma, Urine	Human, Calf, Rat	Harada et al. (1984), Snytnikova et al. (2017a), and Jawor et al. (2019)
Succinate	AH, Cornea, Urine	Human, Calf, Sheep	Snytnikova et al. (2017b), Jawor et al. (2019), and Locci et al. (2019)
Taurine	Heart, Kidney, Skin, Spleen, WAT, AH, VH, Serum, Urine	Human, Calf, Mice, Rabbit, Sheep	Zelentsova et al. (2016), Snytnikova et al. (2017a, b), Jawor et al. (2019), Locci et al. (2019), and Mora-Ortiz et al. (2019)
Threonine	Heart, Plasma, Serum, Urine	Human, Calf, Mice	Snytnikova et al. (2017a), Mora-Ortiz et al. (2019), and Jawor et al. (2019)
Tyrosine	Heart, Kidney, Liver, Plasma, Serum, Skin, Spleen, Urine, WAT	Human, Calf, Mice	Snytnikova et al. (2017a), Mora-Ortiz et al. (2019), and Jawor et al. (2019)

*BCAA* branched chain aminoacids, *AH* aqueous humour, *VH* vitreous humour, WAT white adipose tissue

## Drug-induced biological effects on the metabolome

The main aims of forensic toxicology are both to determine the presence/absence of drugs of abuse in BFs/tissues or in confiscated materials and to investigate the potential biological effects of chemicals in living individuals involved in forensic cases and in post-mortem biological samples collected during an autopsy. While in the first case, the analysis is focused on the identification and quantification of relevant substances either in different biological substrates or in court exhibits, so to confirm their previous assumption or their illegal nature, in the other context the laboratory analysis are an indispensable tool to unravel the cause of death (such as poisoning, drug of abuse toxicity, or accidental/suicidal admixture of medical drugs) either for clinical or legal purposes. A more complex and tough issue, which has not fully addressed yet, is the biological investigation of the metabolic pathways related to an acute or a chronic assumption of one, or more substances, and their potential pathognomonic signature on the human metabolome.

NMR spectroscopy has been widely used for forensic purposes to characterize drugs and their metabolites in human and animal biological matrices (Groombridge 1996; Nicholas et al. 2006; Liu et al. 2010; Santos et al. 2018; Dinis-Oliveira 2019). In the present review, we focused on the ^1^H NMR metabolomic studies dealing with the effects of addictive drugs on the endogenous metabolomic profile, which may be used to address their biological effects in view of the description of a putative substance-specific phenotype. From this point of view, the majority of these studies are related to opioids and alcohol addiction (Table 2 reports the most relevant metabolites related to each of these substances). Animal models are quite suitable to study the mechanism of drug addiction, in order to circumvent the effects of diet, environment, and other external factors on the analysed metabolomic profiles. Both acute and chronic addiction studies have been so far reported (Zaitsu et al. 2016; Steuer et al. 2019).

**Table 2:** Metabolites related to drug/alcohol-induced effects

Metabolite	Sample	Drug	Model	References
Acetate	Liver, Pancreas, Plasma, Serum	Ethanol, Heroin	Human, Mouse, Rat	Bradford et al. (2008), Yoseph et al. (2013), Mostafa et al. (2017), and Ning et al. (2018)
Acetoacetate	Pancreas, Serum, Urine	Ethanol, Heroin, Ketamine	Mouse, Rat	Nicholas et al. (2008), Yoseph et al. (2013), Lu et al. (2016), and Ning et al. (2018)
Acetylcysteine	CNS	Cocaine, MAP, Morphine	Monkey, Rat	Deng et al. (2012), Hu et al. (2012), Li et al. (2012), and Bu et al. (2013)
Alanine	CNS, Serum, Urine	Ethanol	Human, Mouse, Rat	Nicholas et al. (2008), Bradford et al. (2008), Masuo et al. (2009), and Mostafa et al. (2016)
BCAA	CNS, Liver, Urine	Ethanol	Human, Mouse, Rat	Bradford et al. (2008), Masuo et al. (2009), and Vázquez‐Fresno et al. (2012)
Choline	CNS, Liver, Pancreas, Serum	Cocaine, Ethanol, Heroin	Mouse, Rat	Masuo et al. (2009), Li et al. (2012), Yoseph et al. (2013), and Ning et al. (2018)
Citrate	CNS, Serum, Urine	Ethanol, MAP, Morphine	Human, Monkey, Rat	Deng et al. (2012), Bu et al. (2013), Mostafa et al. (2016), and Würtz et al. (2016)
Creatine	CNS, Pancreas, Urine	Cocaine, Ethanol, Ketamine, Morphine	Monkey, Mouse, Rat	Masuo et al. (2009), Deng et al. (2012), Hu et al. (2012), Li et al. (2012), Yoseph et al. (2013), and Lu et al. (2016)
GABA	CNS	Cocaine, Ethanol, MAP, Morphine	Monkey, Rat	Gao et al. (2007), Masuo et al. (2009), Deng et al. (2012), Hu et al. (2012), Li et al. (2012), and Bu et al. (2013)
Glucose	CNS, Liver, Serum	Ethanol, Heroin, Morphine	Monkey, Mouse, Rat	Bradford et al. (2008), Nicholas et al. (2008), Masuo et al. (2009), Deng et al. (2012), and Ning et al. (2018)
Glutamate	CNS, Liver	Cocaine, Ethanol, Morphine	Monkey, Rat	Sharma et al. (2003), Xiang et al. (2006), Gao et al. (2007), Masuo et al. (2009), Deng et al. (2012), Hu et al. (2012), and Li et al. (2012)
Glutamine	CNS, Liver, Serum	Ethanol, Heroin, MAP, Morphine	Human, Monkey, Rat	Xiang et al. (2006), Gao et al. (2007), Masuo et al. (2009), Deng et al. (2012), Hu et al. (2012), Bu et al. (2013), Würtz et al. (2016), and Ning et al. (2018)
Glycine	CNS, Liver, Urine	Cocaine, Ethanol, Ketamine	Human, Rat	Masuo et al. (2009), Li et al. (2012), Lu et al. (2016), and Irwin et al. (2018)
Glutathione	CNS	MAP, Morphine	Monkey, Rat	Deng et al. (2012), Hu et al. (2012), and Bu et al. (2013)
3-OH-butyrate	CNS, Liver, Pancreas, Serum	Cocaine, Ethanol, Heroin	Mouse, Rat	Nicholas et al. (2008), Li et al. (2012), Yoseph et al. (2013), and Ning et al. (2018)
Homocysteic acid	CNS	MAP, Morphine	Monkey, Rat	Deng et al. (2012), Hu et al. (2012), and Bu et al. (2013)
Lactate	CNS, Liver, Serum, Urine	Cocaine, Crack, Ethanol, Heroin, Morphine	Human, Monkey, Mouse, Rat	Sharma et al. (2003), Xiang et al. (2006), Bradford et al. (2008), Nicholas et al. (2008), Deng et al. (2012), Hu et al. (2012), Li et al. (2012), Mostafa et al. (2016), Costa et al. (2019), Irwin et al. (2018), and Ning et al. (2018)
Methionine	CNS	MAP, Morphine	Monkey, Rat	Deng et al. (2012), Hu et al. (2012), and Bu et al. (2013)
Myo-inositol	CNS	Cocaine, Ethanol, MAP, Morphine	Monkey, Rat	Sharma et al. (2003), Xiang et al. (2006), Gao et al. (2007), Masuo et al. (2009), Deng et al. (2012), Hu et al. (2012), Li et al. (2012), and Bu et al. (2013)
NAA	CNS	Cocaine, Ethanol, MAP, Morphine	Monkey, Rat	Sharma et al. (2003), Gao et al. (2007), Masuo et al. (2009), Deng et al. (2012), Hu et al. (2012), Li et al. (2012), and Bu et al. (2013)
Phosphocholine	CNS, Serum	Cocaine, Heroin, MAP, Morphine	Monkey, Rat	Deng et al. (2012), Hu et al. (2012), Li et al. (2012), Bu et al. (2013), and Ning et al. (2018)
Succinate	CNS, Urine	Cocaine, GHB, Ketamine, MAP, Morphine	Human, Rat	Hu et al. (2012), Li et al. (2012), Bu et al. (2013), Lu et al. (2016), and Palomino-Schätzlein et al. (2017)
Succinic acid semialdehyde	CNS	MAP, Morphine	Monkey, Rat	Deng et al. (2012), Hu et al. (2012), and Bu et al. (2013)
Taurine	CNS, Urine	Cocaine, Ethanol, Ketamine, MAP, Morphine	Human, Monkey, Mouse, Rat	Xiang et al. (2006), Gao et al. (2007), Bradford et al. (2008), Masuo et al. (2009), Deng et al. (2012), Hu et al. (2012), Li et al. (2012), Bu et al. (2013), Lu et al. (2016), and Irwin et al. (2018)

*BCAA* branched chain aminoacids, *CNS* central nervous system, *GHB* γ-hydroxybutyric acid, *MAP *methamphetamine

### Effects on the metabolome of several classes of drug of abuse

It is well known that opioids, among which the most relevant are morphine and its derivative heroin (3,6-diacetylmorphine), act on the central nervous systems (CNS). While acute opioid use is described to act on various signal transduction mechanisms, the biological effects of its chronic use, and its influence in the development of tolerance and dependence, are not completely understood yet.

NMR metabolomics was used to evaluate the effects of acute and chronic morphine exposure and morphine withdrawal on rat CNS metabolism (Sharma et al. 2003; Xiang et al. 2006; Gao et al. 2007; Hu et al. 2012; Deng et al. 2012). Brain (*locus coeruleus* and periaqueductal gray) and spinal cord metabolites were measured on untreated rats, acute treated with a single morphine dose, chronic treated for 7 days, and naloxone treated after acute dose (5 animals per group) (Sharma et al. 2003). Chronic morphine treatment induced an increase in lactate and N-acetyl aspartate (NAA) levels in the spinal cord, and this was correlated to the occurrence of anaerobic glycolysis and cellular damage. Glutamate and glutamate-related metabolites (glycine and inositols) decreased in the brain after acute treatment while they increased following chronic treatment due to a compensatory mechanism, which was even more evident during naloxone-precipitated withdrawal.

In a series of papers, the same group of authors extensively studied the effects of chronic morphine treatment and withdrawal on several specific cerebral areas in a rat model (Xiang et al. 2006; Gao et al. 2007; Hu et al. 2012) and in a primate model (Deng et al. 2012). In their first paper, thalamus and somatosensory cortex profiles were investigated (Xiang et al. 2006). 18 rats were exposed to chronic treatment, with a 10 mg/kg morphine dose intra-peritoneally injected twice a day for 10 days, while 6 rats were treated with physiological saline and used as control group. Chronic morphine consumption led to a significant increase of lactate and myo-inositol and a decrease of glutamate in both cerebral areas, whilst aspartate was observed to increase in thalamus and glutamine exhibited a decrease in the somatosensory cortex. During the withdrawal period, the altered metabolites recovered to normal level, except taurine, which showed a significant increase in thalamus. Repeated morphine consumption produced different neurochemical changes involving brain energy metabolism, and activity and transition of neurotransmitters. In the following study morphine-induced modifications were investigated in rat prefrontal cortex (PFC) and hippocampus (Gao et al. 2007). The drug administration protocol was the same as previously, but both the treatment and the withdrawal periods were variable. Several region specific metabolic alterations were identified in response to morphine treatment and during the process of morphine discontinuation. In particular, after 10 days of chronic morphine treatment, a significant increase of γ-aminobutyric acid (GABA) and a decrease of glutamate were observed in PFC, whilst both metabolites decreased significantly in hippocampus, compared to controls. Moreover, an increase of glutamine and myo-inositol and a decrease of taurine and NAA were observed in hippocampus. After 3 days of withdrawal, the metabolite levels were comparable to those of the control group, indicating an almost complete recovery, suggesting that the morphine-induced CNS adaptive reaction is reversible upon withdrawal. Then, the study was extended to brain hippocampus, *nucleus accumbens* (NAc), PFC, and *striatum* (Hu et al. 2012). 24 rats were assigned to 4 groups: three groups received increasing intra-peritoneal morphine doses twice a day for 14 days, the control group was treated with saline solution for 14 days. Following morphine administration, the three groups of rats received methadone, clonidine, and saline, respectively, while the control rats did not receive anything. Perturbation of energy metabolism, amino acid metabolism, and neurotransmitters was observed and interpreted as the result of oxidative stress and adaptive mechanism in reaction to chronic morphine use. Differential disturbances of the neurotransmitters glutamine, glutamate, and GABA were observed in the examined cerebral regions. Significant changes were also observed in NAA, taurine, and phosphocholine, and they were associated to oxidative stress and membrane disruption caused by morphine. Lactate was observed to increase in the four regions of dependent rats. The TCA intermediates α-ketoglutarate and succinate showed significant differential changes in the four regions. Remarkably, the use of methadone and clonidine led to the recovery of the brain metabolic disturbances. The same approach was applied to the study of the modifications induced in brain hippocampus and PFC of rhesus monkeys (Deng et al. 2012). The authors essentially confirmed their previous results observing modifications of energy metabolism, membrane, and neurotransmitters after chronic morphine exposure, and complete metabolic recovery following pharmacotherapies with methadone and clonidine.

The effects of another addictive opioid, i.e. heroin (3,6-diacetylmorphine), were investigated using a heroin self-administration rat model (Ning et al. 2018). The authors focused their attention on the serum metabolic modifications occurring upon reinforcement (heroin reintroduction) after abstinence. To this aim, 12 rats were trained to self-administer heroin for 9 days (5 of them were successively excluded), they were left in abstinence for 14 days, then they were reinforced to heroin and blood samples were collected. Several metabolites resulted altered after heroin reinforcement compared with those in the control group. In particular, the increase of glucose and the decrease of the intermediates of both glycolysis and TCA cycle (lactate, pyruvate, and fumarate) suggested that energy production was considerably disturbed. The high levels of 3-hydroxybutyrate and acetoacetate suggested activation of ketogenesis from fatty acids as an alternative energy source for the brain. The increase of neurotransmitters precursors such as choline, phenylalanine, and glutamine indicated a toxic disturbance in the substrate supply by heroin.

Changes in cerebral metabolites following cocaine treatment were also reported (Li et al. 2012). Cocaine is a psychostimulant, which gives dependence and persists for very long times after withdrawal. NAc and striatum metabolomic profiles were monitored in a murine model subjected to single and repeated cocaine treatments. Significant region specific modifications were observed in metabolites involved in neurotransmission, oxidative stress, mitochondria dysregulation, and membrane disruption. After a single cocaine treatment, metabolites like NAA, lactate, choline, phosphocholine, and myo-inositol were altered in both cerebral areas. The main modifications identified after repeated treatment were the increase of the neurotransmitters glutamate and GABA and the increase of NAA in NAc and *striatum*. Moreover, the increase of creatine and taurine was observed in NAc, whereas the increase of taurine and lactate and the decrease of creatine were observed in striatum. The alterations of lactate and NAA could reflect mitochondria dysregulation caused by cocaine. The behaviour of choline and phosphocholine could be related to membrane disruption.

^1^H NMR metabolomics was also used to discriminate crack dependent users from healthy individuals based on the serum metabolomic profile (Costa et al. 2019). The reduction of carnitines and acylcarnitines and the accumulation of histidine were considered indicative of a loss of energetic metabolism in the brain due to their deprived nutritional behaviour and of an impaired histamine biosynthesis. Higher levels of lactate were associated with an increased synaptic activity and the increase of phenylalanine and tyrosine was related to an altered dopamine biosynthesis.

The effects of methamphetamine (MAP) exposure on the neuronal metabolome were investigated in a murine model (Bu et al. 2013). 10 rats were subjected to subcutaneous MAP injections, twice a day for 7 days. NAc, PFC, and hippocampus metabolites were analysed and compared with control samples. Many metabolites identified in the examined cerebral areas contributed to the discrimination of MAP treated and control samples. A significant decrease in the neurotransmitters glutamate, glutamine, and GABA, and in the antioxidant glutathione was observed in the three cerebral areas. Decrease of acetylcysteine in hippocampus and NAc, and of taurine in NAc and PFC was observed. All these metabolites could play a crucial role in MAP-induced behavioural sensitization. Furthermore, NAA, a marker of neuronal viability, decreased in the three areas, while phosphocholine increased in NAc and PFC. TCA cycle intermediates decrease was observed in all the MAP treated samples. The elevation of myo-inositol in hippocampus and PFC could reflect glial activation and proliferation. The results globally suggested that MAP induced alterations in neurotransmission, oxidative stress, membrane disruption, and energetic alterations in all the studied cerebral regions.

The effect of short-term ketamine treatment on the urinary metabolomic profile was investigated in a rat model (Lu et al. 2016). Ten rats received increasing intraperitoneal doses of ketamine over a period of 9 days, while ten control rats received saline. Ketamine exposure led to significant changes in metabolites related to antioxidant and energy metabolism, including acetoacetate, succinate, 1,3,7-trimethyluric acid, 1,3-dimethyluric acid, creatine, and taurine.

NMR metabolomics was employed to detect and identify potential biomarkers related to exogenous γ-hydroxybutyric acid (GHB) in human urine and serum samples (Palomino-Schätzlein et al. 2017). GHB is known as “liquid ecstasy” and is frequently associated with cases of drug-facilitated sexual assault. The study was conducted on 12 healthy volunteers. Urine and blood samples were taken before and at different time-points after GHB administration. The results indicated that exogenous GHB is metabolized to succinate and glycolate similarly to what happens to endogenous GHB. Glycolate was still altered 20 h post-dose and was suggested as a candidate biomarker of GHB abuse.

Despite the experimental designs of the reviewed studies are very heterogeneous, some general considerations can be drawn, being the energetic pathways mainly involved. Lactate increase is constantly observed, suggesting that the effect of the different drugs leads to an impairment of the glycolysis and a shift toward anaerobic conditions. Perturbation of the TCA cycle intermediates together with modifications in glutamine-glutamate levels are indicative of a TCA cycle derangement. If so, ^1^H NMR should be devoted not to the identification of the drug or its metabolites on several BFs—which are, up to now, routinely and efficiently analysed using GC- and/or LC–MS—but to the analysis of the metabolomic profiles related drugs of abuse assumption. Of foremost forensic interest, ^1^H NMR metabolomics may be helpful in the identification of the cause of death in lethal cases when an acute consumption of a drug of abuse is suspected. The mere detection of relevant substances in BFs—although suggestive of the manner of death whenever their concentrations are included in the range of lethal doses available in published databases—is not per se always sufficient to establish the causal relationship between assumption and death. The concentrations detected in the major BFs of a corpse may be observed even in living individuals who are under the effects of the same substance. An objective snapshot of the metabolomic perturbation at the moment of death may be of some help in assigning the cause of death to the effects of acute—or, to better say, acute in a chronic context—assumption of a drug of abuse. If these results will be confirmed in independent researches and in different laboratories, the relevant modifications in serum/plasma or urine metabolome, related to one or more classes of recreational drugs, may be employed even to monitoring the individual compliance with rehabilitation protocols. In these cases, it is well-known that abusers may result negative to the routine screening analysis—only because they refrain from assuming the drug in view of a previous established control—and a tool to ascertain their ‘chronic’ condition may be of help in the identification of true negative individuals from ‘temporary’ ones. In the forensic scenario, the search for alternative biological matrices able to store memory of previous intake is a hot topic, and hairs and other keratinised matrices are commonly employed to prove the presence/absence of the non-metabolised molecule or of its by-products going back to several days from the last assumption. But this evidence gives no information on the biological effects of the substance, while a metabolomic profile may be able to describe even its long-lasting perturbation on the metabolism. The combined use of these two approaches may give a ‘holistic’ picture of the abuser, so to let the judge take a decision on a sounder scientific ground.

### Alcohol effects on the metabolome

Several studies focused on the pathophysiological consequences of acute and chronic alcohol consumption on the human or animal metabolome (Bradford et al. 2008; Nicholas et al. 2008; Masuo et al. 2009; Fernando et al. 2010; Vázquez‐Fresno et al. 2012; Yoseph et al. 2013; Mostafa et al. 2016, 2017; Würtz et al. 2016). NMR profiles of urine, serum, or tissues from murine models or serum from human volunteers were investigated, as described hereafter.

Rat blood, liver, and brain biochemical changes following either a single dose of ethanol or a 4 days binge-ethanol protocol were monitored (Nicholas et. al. 2008). Before starting the chronic alcohol administration, half of the rats were pre-treated with butylated hydroxytoluene, an antioxidant that shows a protective role against ethanol-induced brain damage. With respect to control samples, both acute and binge-ethanol treatments induced decrease of glucose, lactate, and alanine and increase of acetate in serum and in liver, while increase of acetoacetate was observed only in serum. Moreover, the chronic intake led to an increase in 3-hydroxybutyrate and a decrease in betaine in liver. Pre-treatment with butylated hydroxytoluene increased betaine levels and decreased those of ethyl glucuronide in liver. No changes were found in brain metabolites after a single dose of ethanol. They proposed a new mechanism that compensates the lack of gluconeogenesis consequent to ethanol treatment.

Metabolomic analysis of mice urines and liver extracts were conducted to monitor the biochemical changes associated to the disease progression towards the alcohol-induced liver injury (Bradford et al. 2008). Mice were exposed to increasing doses of alcohol (from 7 g/kg/day to 21 g/kg/day) for 36 days. A sixfold increase in liver injury scores (necrosis, inflammation, and steatosis) was observed. In liver high levels of lactate and alanine indicated activation of hypoxia and glycolysis and high levels of linoleic acid (a precursor of prostaglandins) was consistent with liver inflammation. Alcohol-induced urinary increase in lactate, n-acetylglutamine, n-acetylglycine, and decrease in taurine. These metabolites were considered potential markers of chronic alcohol consumption and consequent oxidative stress.

The effects of chronic administration of Sake, a Japanese beverage containing 15% of alcohol, on rat brain and liver were investigated (Masuo et al. 2009). Five rats were given free access to Sake for 12 months and brain and liver metabolomic modifications were compared with those of five control animals. The results indicated an attenuation of the mitochondrial function in the liver, whilst the analysis of brain revealed a significant increase in valine, arginine, ornithine, alanine, glutamine, and choline and a decrease in isoleucine, NAA, taurine, glutamate, and GABA. The authors seem to share the same biological explanation proposed by Nicholas et al. (2008) concerning the “empty calorie” phenomenon of ethanol.

In another study, 344 rats were fed with a chronic alcoholic (5%) diet for 1 month. Control rats were pair-fed an equivalent amount of maltose-dextrin (Fernando et al. 2010). After 1 month, animals were sacrificed, plasma and hepatic samples were collected, and lipidomic analysis was performed. Ethanol consumption induced alteration in the metabolism of cholesterol, triglycerides, and phospholipids that could contribute to the development of fatty liver. Fatty acids and triglycerides were found increased while phosphatidylcholine decreased in liver. In plasma samples, both fatty acids and phosphatidylcholine were decreased. The authors concluded that liver and plasma lipid profiles may be important for detecting early stage of alcoholic liver disease.

The pancreatic metabolome was analysed in mice fed ad libitum with increasing concentration of alcohol for two weeks (Yoseph et al. 2013). Increased levels of acetate, adenosine, xanthine, acetoacetate, 3-hydroxybutyrate and betaine and decreased levels of cytidine, uracil, fumarate, creatine phosphate, creatine, and choline were detected upon chronic alcohol intake.

An untargeted NMR metabolomic study was conducted on wine consumers male volunteers at high cardiovascular risk (Vázquez‐Fresno et al. 2012). The participants followed alternatively three different dietary interventions for 4 weeks: dealcoholised red wine, alcoholised red wine, and gin. After each intervention, 24-h urine samples were collected and analysed. After analysing the global urinary profile, only the relevant metabolites were selected. Mannitol was associated to diet and tartrate to wine intake. An increase in 3-methyl-2-oxovalerate suggested a possible up-regulation of the first step of branched-chain amino acid catabolism after wine consumption. Moreover, alterations in the gut microbiota metabolites 4-hydroxyphenylacetate and hippurate were identified.

Finally, ^1^H NMR metabolomics was used to discriminate between alcohol-dependent and non-alcohol dependent alcohol drinkers and controls (Mostafa et al. 2016, 2017). Urine and plasma samples were collected from 30 alcohol-dependent (AD) individuals, 54 social drinkers, and 60 controls. While individuals of the two alcohol groups were all males, the control group was formed by both males and females and was not completely matched for age with the alcohol dependent group. Moreover, the enrolled individuals belonged to different ethnicity. These aspects could represent serious confounding factors, as underlined by the same authors. PCA analysis indicated that social drinkers and control samples were randomly distributed and separated from the AD group, so they were combined in a single group for comparison with AD samples in supervised OPLS-DA analysis. OPLS-DA confirmed that AD group was significantly discriminated from the other with high sensitivity and specificity. Urinary metabolites highly correlated with the AD group were cis-aconitic acid, citric acid, alanine, lactic acid, 1,2-propanediol and 2-hydroxyisovaleric acid. All these metabolites except 2-hydroxyisovaleric acid are normally present in urine but in the chronic alcohol consumption group their concentrations were markedly increased. The presence of 2-hydroxyisovaleric acid in chronic consumers was attributed to an increased lactic acidosis. In plasma samples they identified acetic acid and propionic acid associated to chronic alcohol consumption. The author concluded with the need of biomarker validation and generalization of these results to other population.

A meta-analysis was conducted on three cohorts of 9778 young Finnish adults with different habitual usage of alcohol (Würtz et al. 2016). Alcohol intake induced increase of serum monounsaturated fatty acids and high-density lipoprotein, and decrease of omega-6 fatty acids, glutamine, and citrate, very low-density lipoprotein, indicating an increased cardiometabolic risk.

A 4-arms crossover experiment on the effect of acute alcohol consumption was performed on healthy moderate-drinking young men (Irwin et al. 2018). Urine metabolomic profile was investigated in the four groups receiving alternatively: (a) a benzoic acid-containing flavoured water vehicle; (b) a defined acute dose of alcohol; (c) a Nicotinamide Adenine Dinucleotide (NAD) supplement followed by the vehicle alone; and (d) a NAD supplement together with vehicle and alcohol. Acute alcohol consumption induced an overproduction of reducing equivalents and NAD^+^ depletion. The former phenomenon was related to the up-regulation of the lactic acid metabolism, while the latter was supposed to be a consequence of the down-regulation of both purine catabolism (being alcohol able to decrease the conversion of hypoxanthine to xanthine) and osmoregulation release (increase of trimethylamine N-oxide and sorbitol). Hepatocyte redox homeostasis resulted to be greatly perturbed, being urinary sorbitol excretion an intriguing potential marker.

Even in this experimental context, the promising overall results so far obtained seem to be faded by the unclear design of experiments, the heterogeneity of primary and secondary endpoints, the polymorphic choice of BFs/tissues and animal model, the inner difficulties to discriminate metabolomic effects of an acute from a chronic assumption and, even more, of acute ones in chronic dependent consumers. As it can be evinced from data in Table 2, a call for multicentric studies with a very large number of samples/individuals should be performed, in order to assess how informative are the identified metabolites as biomarkers or which is the most useful tissue to analyse to confirm or exclude a pathological diagnosis. Moreover, it seems to be mandatory to draw robust experimental designs, to be firstly investigated on animal models and then translated into a human setting, and this cannot be postponed any longer. Despite some molecules are constantly found—as a *fil rouge*—in different studies focusing on a shared topic, at the moment, none of them may be individually proposed as a diagnostic tool, residing the advantage of NMR metabolomics in the possibility of comparing global profiles.

## Conclusions

NMR metabolomics is a versatile technique that can be applied to the study of several biological fluids and tissues. Relying on the endogenous metabolomic profiling, it allows to study a biological system, monitor, and possibly predict the modifications associated to different forensic conditions as the ones described in this review. The analysis of the post-mortem metabolomic changes, for example, indicates a correlation between PMI and the metabolic profiling, paving the way for developing new predictive tools for the PMI estimation.

Considering that the metabolome is strongly influenced by several external parameters, such as for example diet, environmental conditions, and pathological states, animal models are largely used since they allow to carefully control the experimental conditions and detect the modifications solely related to a specific induced stimulus. In addition, the use of animal models offers the possibility of using control animals, which in the case of human studies is often problematic for ethical reasons. Animal models are very suggestive for translation to humans, and, once specific profiles or potential biomarkers are identified, they may help in building causative hypothesis to be tested.

Despite its low intrinsic sensitivity, NMR does not require sample extraction procedures, it is robust, highly reproducible, non-selective, and non-invasive. These features let NMR be a powerful analytical tool for studying biological systems, identifying metabolic signatures, and extracting, in combination with multivariate statistical analysis, relevant information on the effects of specific stimuli. A major limit of NMR metabolomics may be the availability of a suitable sample volume for forensic analysis. While this would not be a problem when samples are collected from living individuals or corpses, it could be a concern in the case of BF identification from stains. Moreover, we are also aware that NMR is an expensive equipment, nowadays easily available in the academic context, but not always accessible to forensic laboratories outside the universities. However, it has the potential of giving information on global metabolomic modifications without any a priori knowledge and therefore it could pave the way for more specific, targeted and also more sensitive analysis to be conducted via GC or LC–MS which at the best of our knowledge is usually available in a forensic laboratory.

Although NMR metabolomics could help to reach a personalized approach in several forensic scenarios, it seems to us that there is still a long way from bench to court-side.

Notwithstanding the growing use of NMR metabolomics in various fields of forensic science, its potential in the forensic pathology scenario has not been completely exploited. A very recent work published in the literature opens the way for differential diagnosis of asphyxial deaths (Varvarousis et al. 2017) suggesting that more efforts should be also made in this field.
